# Specific suppression of long terminal repeat retrotransposon mobilization in plants

**DOI:** 10.1093/plphys/kiac605

**Published:** 2022-12-30

**Authors:** Anna Brestovitsky, Mayumi Iwasaki, Jungnam Cho, Natthawut Adulyanukosol, Jerzy Paszkowski, Marco Catoni

**Affiliations:** The Sainsbury Laboratory, University of Cambridge, Cambridge CB2 1LR, UK; The Sainsbury Laboratory, University of Cambridge, Cambridge CB2 1LR, UK; Department of Plant Biology, University of Geneva, Geneva CH-1211, Switzerland; The Sainsbury Laboratory, University of Cambridge, Cambridge CB2 1LR, UK; CAS Center for Excellence in Molecular Plant Sciences, Chinese Academy of Sciences, Shanghai 200032, China; The Sainsbury Laboratory, University of Cambridge, Cambridge CB2 1LR, UK; The Sainsbury Laboratory, University of Cambridge, Cambridge CB2 1LR, UK; The Sainsbury Laboratory, University of Cambridge, Cambridge CB2 1LR, UK; School of Biosciences, University of Birmingham, Birmingham B15 2TT, UK; Institute for Sustainable Plant Protection, National Research Council of Italy, Torino 10135, Italy

## Abstract

The tissue culture passage necessary for the generation of transgenic plants induces genome instability. This instability predominantly involves the uncontrolled mobilization of LTR retrotransposons (LTR-TEs), which are the most abundant class of mobile genetic elements in plant genomes. Here, we demonstrate that in conditions inductive for high LTR-TE mobilization, like abiotic stress in Arabidopsis (*Arabidopsis thaliana*) and callus culture in rice (*Oryza sativa*), application of the reverse transcriptase (RT) inhibitor known as Tenofovir substantially affects LTR-TE RT activity without interfering with plant development. We observed that Tenofovir reduces extrachromosomal DNA accumulation and prevents new genomic integrations of the active LTR-TE *ONSEN* in heat-stressed Arabidopsis seedlings, and *transposons of O. sativa 17* and *19* (*Tos17* and *Tos19*) in rice calli. In addition, Tenofovir allows the recovery of plants free from new LTR-TE insertions. We propose the use of Tenofovir as a tool for studies of LTR-TE transposition and for limiting genetic instabilities of plants derived from tissue culture.

## Teaser

Plant tissue culture supplemented with the inhibitor of reverse transcriptase Tenofovir prevents mobilization of retrotransposons and increases the genetic stability of regenerated plants.

## Graphical Abstract

**Figure:**
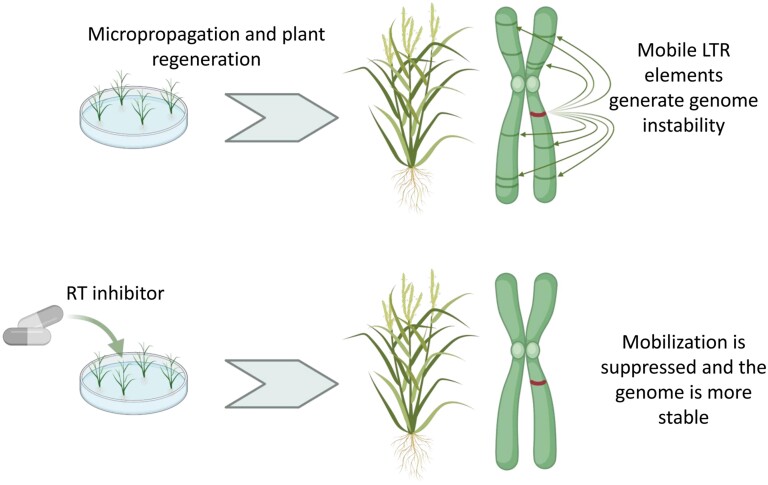

## Introduction

Plant tissue culture is commonly used in agriculture for micropropagation of genetically homogeneous and disease-free plant material, for genetic transformation, and production of improved plant varieties (Hussain et al., 2012). This technique is known to induce somaclonal variation, consisting of both genetic and epigenetic unintentional alterations, which are impossible to predict and therefore are generally undesirable in the final regenerated plants (Larkin and Scowcroft, 1981; McClintock, 1984; Wang et al., 2013). Somaclonal variation during tissue culture can be generated by various mechanisms, however, it is been well documented that one of the most common genetic alterations observed is the uncontrolled movement of long terminal repeat (LTR) transposable elements (LTR-TEs or retrotransposons) (Azman et al., 2014; Grandbastien, 2015; Bednarek and Orłowska, 2020). LTR-TEs are endogenous genetic elements abundant in plant genomes, which can extrachromosomally replicate their DNA copies and integrate them into new chromosomal locations.

Under normal physiological conditions, the activity of LTR-TEs is suppressed by epigenetic mechanisms. However, the process of tissue culture can be perceived as a genomic stress and causes TEs activation and their mobilization, leading to uncontrolled mutagenic effects in regenerated plants (Hirochika et al., 1996; Takeda et al., 2001; Fukai et al., 2010; Evrensel et al., 2011; Sabot et al., 2011; Wang et al., 2013). For example, somaclonal variation has been well studied in rice (*Oryza sativa*) (Jiang et al., 2003; Kikuchi et al., 2003; Wang et al., 2009, 2013), and it is known that in vitro propagation of undifferentiated callus tissue induces mobilization of several rice LTR-TEs (Hirochika et al., 1996; Sabot et al., 2011). Consequently, each rice plant regenerated from such calli displays a variety of new retrotransposon insertions in multiple chromosomal locations (Sabot et al., 2011). Among rice LTR-TEs, *Tos17* is the most active in tissue culture, and it is able to insert into 30 new chromosomal positions in a single regenerated plant (Hirochika et al., 1996; Lanciano et al., 2017). Most importantly, *Tos17* tends to transpose into coding regions of genes, potentially interfering with their function (Miyao et al., 2003; Piffanelli et al., 2007). Considering that the locations and the precise effects of a transposon insertion cannot be easily predicted, newly generated LTR-TE insertions represent a serious threat to the genetic stability and safe use of transgenic rice varieties that are obtained through in vitro regeneration processes.

For their extrachromosomal propagation, LTR-TEs use self-encoded reverse transcriptase (RT) to replicate their genome, using RNA as an intermediary template (Lisch, 2013). This process mirrors the replication strategy of mammalian retroviruses, like human immunodeficiency virus (HIV), which encodes for an analogous RT enzyme (Levy, 1989). Among possible treatments for these diseases, several drugs with RT inhibitory action have been developed and used as efficient anti-HIV agents (De Clercq, 2009). Nonetheless, although these drugs were found to be able to suppress retrotransposon activity in mammalian cells (Jones et al., 2008; Banuelos-Sanchez et al., 2019), they have never been tested as possible inhibitors of RT enzymes encoded by plant LTR-TEs, and thus are a possible approach to protect plant genomes against uncontrolled retrotransposition.

Here, we examined RT inhibitors for their possible activity to suppress LTR-TEs mobilization in two plant species, employing well-documented conditions provoking retrotransposition. Specifically, we show that the application of Tenofovir, a nucleotide RT inhibitor, prevents mobilization of the heat-inducible LTR-TE known as *ONSEN* in Arabidopsis (*Arabidopsis thaliana*), without substantially impacting gene expression and plant development. Moreover, we show that the application of Tenofovir to rice callus cultures precludes new LTR-TE insertions in regenerated plants. These results suggest that Tenofovir could be widely used in tissue culture procedures, resulting in regeneration of genetically more uniform plant material.

## Results

### Tenofovir efficiently suppresses *ONSEN* extrachromosomal DNA accumulation and its retrotransposition in Arabidopsis without affecting plant growth

To screen for the most effective drug to inhibit the activity of retrotransposon encoded RT in plants, we used a system based on the environmentally controlled mobilization of the LTR transposon *ONSEN* in *A. thaliana. ONSEN* is activated in response to heat stress and mobilizes in plants mutated in NPRD1, which is the main subunit of the plant-specific RNA polymerase IV (Ito et al., 2011). In heat-stressed *nprd1* seedlings, *ONSEN* activation can be easily detected by measuring its increased copy number, which is induced by the accumulation of its reverse transcribed extrachromosomal DNA (ecDNA) and by integrations into new chromosomal loci. We germinated *nprd1* seedlings in media containing two concentrations (1 and 10 µM) of well-characterized RT inhibitors. This included the nucleoside RT inhibitors Zidovudine, Stavudine, and Lamivudine; the nucleotide RT inhibitor Tenofovir; and the non-nucleoside RT inhibitor Nevirapine (De Clercq, 2009). Using quantitative polymerase chain reaction (qPCR), we measured the accumulation of *ONSEN* ecDNA induced by heat stress *in planta* for each drug concentration, compared to the levels accumulated in seedling grown without RT inhibitors.

**Figure 1:**
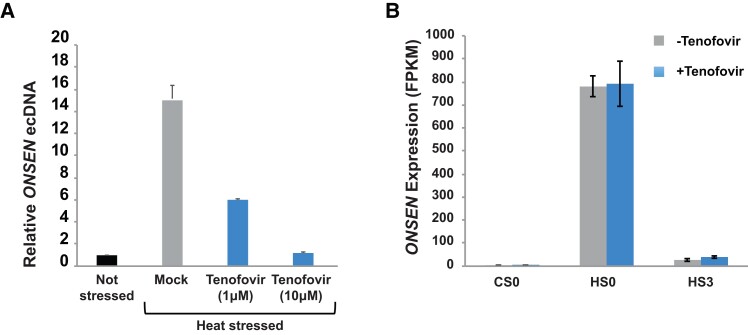
Inhibition of *ONSEN* ecDNA in *A. thaliana* using tenofovir. A, Quantification of *ONSEN* DNA copy number by qPCR in heat-stressed *A. thaliana nprd1* mutant seedlings germinated in the presence of Tenofovir (1 and 10 µM). Copy number is displayed relative to the genomic *ONSEN* copies. Unstressed mutant seedlings (marked as “Not stressed”) and heat-stressed seedlings without drugs (Mock) were used as controls. Each bar represents the mean of three technical replicates; ±Sd marked by error bars. B, Quantification of changes in *ONSEN* mRNA accumulation as a consequence of heat stress in *A. thaliana nprd1* seedlings, in the absence or the presence of 10 µM Tenofovir in the medium. Each bar represents the mean of three biological replicates; ±Sd marked by error bars. CS0 = not stressed plants. HS0 = after heat stress. HS3 = three days after stress was applied.

We observed that Tenofovir at 1 µM in the medium was able to reduce *ONSEN* ecDNA to approximately 50%, while ecDNA accumulation was barely detectable when 10 µM concentration of Tenofovir was used (Figure 1A). In this screening attempt, we did not observe substantial effects in reducing *ONSEN* ecDNA levels for the other tested drugs (Supplemental Figure 1), and therefore we focused on Tenofovir in all subsequent experiments.

To determine the specificity of Tenofovir activity, we investigated the possible effects induced by this drug on Arabidopsis gene expression by genome-wide RNA-seq. We compared the expression profiles of wild-type seedlings grown in control media and media supplemented with 10 µM Tenofovir. Experiments were performed under standard growth conditions, with or without heat stress, and after 3 d of recovery following heat stress (Supplemental Figure 2A and Supplemental Table 1). First, we focused our attention on *ONSEN* mRNA level, which is known to accumulate during heat stress (Ito et al., 2011; Cavrak et al., 2014). We observed that the presence of Tenofovir in the media did not alter the levels of *ONSEN* transcripts induced in response to elevated temperatures (Figure 1B). Therefore, while Tenofovir inhibited accumulation of *ONSEN* ecDNA (Figure 1A), it did not affect the increase in *ONSEN*'s mRNA levels after application of heat stress (Figure 1B).

**Figure 2:**
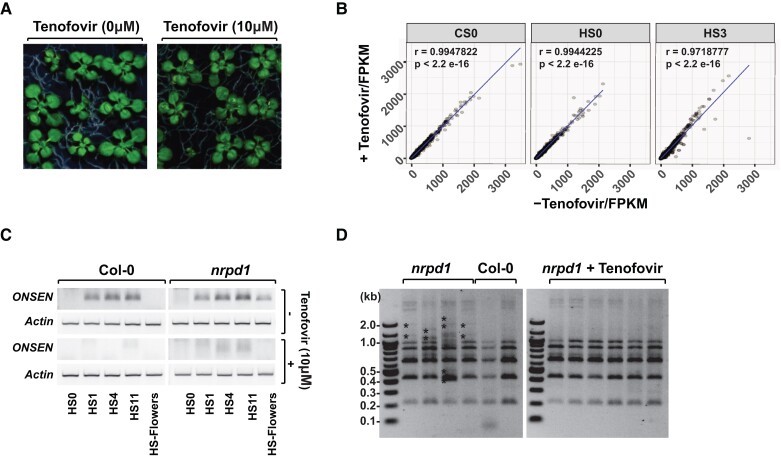
Tenofovir prevents *ONSEN* ecDNA accumulation and its genome integration without substantially affecting plant growth and response to heat. A, Representative pictures of mock-treated control plants (0 µM) and Tenofovir-treated plants (10 µM) grown in plates for 14 days at 21°C, displaying absence of visible alterations induced by Tenofovir. B, Scatter plots summarizing correlation of gene expression in plant samples grown in the absence or the presence of Tenofovir (10 µM). The data are from not stressed plants (CS0), plants collected after heat stress (HS0), and three days after the stress was applied (HS3). Each dot represents a single gene. Pearson correlation was used to assess the significance of the gene expression differences (r and *P*-value are indicated). C, Detection (by inverse-PCR) of circular DNA forms of *ONSEN* in Col-0 and *nrpd1-3* plants, grown in the absence or the presence of Tenofovir (10 µM). The amplification of *ACT7* (AT5G09810) was used as a loading control. The conditions tested include stressed plants (HS0); plants collected after 1, 4, and 11 days of recovery from the stress (HS1, HS4, and HS11, respectively); and flower tissue obtained from recovering plants at flowering time, 20 days after the stress (HS-flowers). D Transposon display performed on genomic DNA from single *nprd1* mutant seedlings from the seed progeny of Arabidopsis stressed plants in the presence or the absence of Tenofovir (10 µM). Samples from Col-0 heat-stressed parents were used as control. New insertions are marked with stars (*).

The seedlings grown in the presence of Tenofovir have not shown directly observable alterations in term of their growth and development for all life cycle (Figure 2A), all plants flowered and produced seeds at comparable time, and their progenies developed normal. To determine possible hidden effects of Tenofovir on gene expression, we applied hierarchical clustering of the whole-plant transcriptomics obtained from our various experimental samples. We observed that regardless of the presence of Tenofovir, the replicates of all the samples clustered only in accordance with their heat treatments, indicating that the effect of Tenofovir is negligible in relation to the effect of heat stress (Supplemental Figure 2B). Additionally, the expression profiles of seedlings grown in the absence or in the presence of Tenofovir under control conditions, heat stress, and after recovery correlate, respectively, at 99%, 99%, and 97% (Figure 2B, Supplemental Table 2), indicating very similar mRNA profiles. A similar result was obtained when we compared specifically TE expression, which was found to correlate at 98% for samples grown in the absence or in the presence of Tenofovir for all three conditions analyzed (Supplemental Figure 2C, Supplemental Table 3).

Our differential expression analysis identified only 80 genes with more than two times difference in the level of mRNA that can be attributed to the Tenofovir treatment. Of these, 44 differentially expressed genes (DEGs) were found in control conditions, 13 DEGs were found in the heat stress condition, and 26 DEGs after recovery from heat stress, with only three DEGs identified in more than one condition (Supplemental Table 4). Within the DEGs identified in the control samples grown in the presence and absence of Tenofovir (CS0+/−T), there was an enrichment of “Plant-type cell wall organization” (seven genes, false discovery rate (FDR) = 0.00054) and “Root morphogenesis” (five genes, FDR = 0.038) genes (Supplemental Table 5). There was no significant gene ontology (GO) enrichment in DEGs due to Tenofovir treatment after heat stress (HS0+/−T), while 14 DEGs in recovery samples (HS3+/−T) had GO related to “Response to stress, heat, and bacterium” (FDR = 0.00028) (Supplemental Table 6).

The production of ecDNA is a necessary step for transposition of LTR elements. New *ONSEN* insertions were observed in the progeny of heat-stressed *nprd1* in previous studies (Ito et al., 2011), indicating that *ONSEN* ecDNA persists during plant development and can integrate in flower germ cells of mutant plants. Therefore, we measured by qPCR the ecDNA accumulation in both wild type and *nprd1* mutant heat-stressed seedlings at 0, 1, 4, and 11 days following the heat stress, as well as in the flowers of fully developed plants. We observed that the reduction in *ONSEN* ecDNA induced by Tenofovir application was still significant at 11 days after the heat stress (Supplemental Figure 2D). Considering that qPCR detects ecDNA formation by measuring the number of all *ONSEN* DNA copies (including the original copies integrated in the genome), this approach seems to be not sensitive enough to detect low levels of residual ecDNA persisting in flowers of plants stressed at the seedling stage. Therefore, to detect *ONSEN* ecDNA in plants at flowering stage we used inverse primers to amplify selectively *ONSEN* circular DNA molecules. This circular DNA is considered to be a byproduct of LTR-TE transposition with exclusively extrachromosomal nature (Lanciano et al., 2017). We observed that Tenofovir application can abolish or strongly reduce the production of circular ecDNA in Col-0 and *nprd1* mutant seedlings (Figure 2C). Interestingly, the circular ecDNA was also not detectable in flowers of Tenofovir-treated *nprd1* seedlings, potentially suggesting that the drug application could limit *ONSEN* transposition in the progeny of treated plants. To test this hypothesis directly, we investigated the presence of new *ONSEN* insertions in the progeny of heat-stressed *nprd1* plants by transposon display. While the progeny of control plants displayed several new *ONSEN* insertions, these were not detected in the progeny of plants treated with Tenofovir (Figure 2D).

Collectively, these results indicate that the application of Tenofovir can efficiently suppress *ONSEN* RT activity, inhibiting both *ONSEN* ecDNA formation and subsequent transposition, with negligible effects on gene or TE expression and Arabidopsis development.

### Rice plants regenerated from Tenofovir-treated calli are free from new LTR-TE insertions

**Figure 3:**
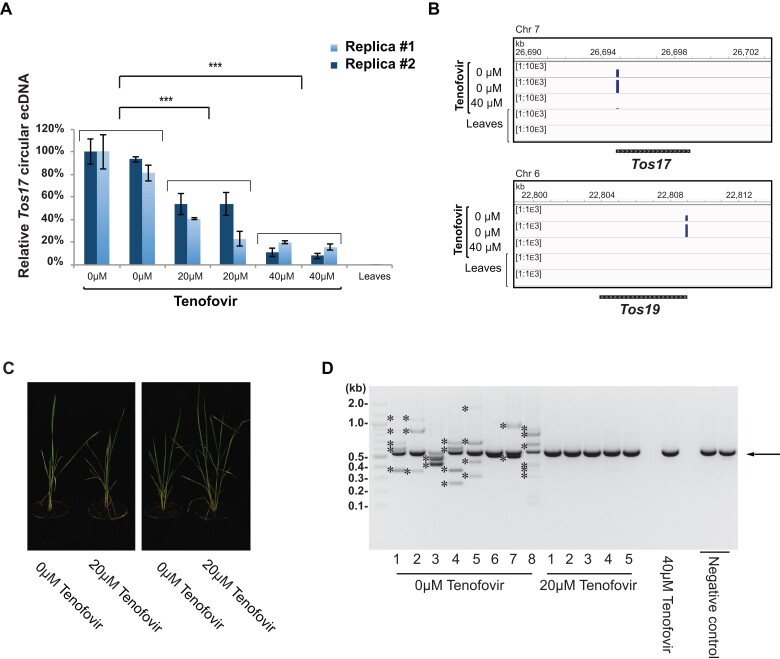
Rice plants regenerated in the presence of Tenofovir are free from *Tos17* new insertions. A, Quantification by qPCR of circular ecDNA of *Tos17* accumulating in rice calli induced in media supplemented with 0, 20, and 40 µM Tenofovir. The relative copy number of ecDNA was calculated using Ubiquitin gene as a reference. The values displayed are relative to *Tos17* DNA levels in samples from Tenofovir-untreated callus. The DNA from leaves was used as a negative control. Two biological replicates are displayed. Each bar represents the mean of three technical replicates; ±Sd marked by error bars. *P*-values were calculated by a one-tailed Student's *t* test. *** = *P* 1 × 10^−4^. B, Read coverage plots obtained from the ALE-seq of rice callus induced in the absence (0 µM) or the presence (40 µM) of Tenofovir, mapped at *Tos17* and *Tos19* genomic loci. DNA extracted from leaves was used as a negative control. The coverage range is indicated in square brackets. C, Two representative examples of rice plants regenerated from calli propagated in the absence or the presence of Tenofovir (0 and 20 µM) displaying similar development and architecture. D, Transposon display of *Tos17* in plants regenerated from calli in the absence or the presence of Tenofovir (0, 20, and 40 µM). The most evident band common to all plants corresponds to the original *Tos17* copy in the reference genome (indicated with arrow). Bands marked with stars (*) correspond to new *Tos17* insertions. The number of new *Tos17* insertions in Tenofovir-treated and control plants has been found to be significantly different (*P*-value = 0.0067, Mann–Whitney U test). DNA extracted from leaves of naturally propagated plants was used as a negative control.

To directly test if the application of Tenofovir in tissue culture of crop plants prevents LTR transposon mobilization, we tested the use of this drug during propagation of rice calli, which is a condition where multiple LTR retrotransposons can be mobilized and lead to genetic variation in the regenerated plants (Hirochika et al., 1996). We induced rice calli in vitro from scutellum tissue of mature seeds (Nishimura et al., 2006), using either a standard medium, or the medium containing Tenofovir at 20 and 40 µM (Supplemental Figure 3A). We observed that the presence of Tenofovir in the medium strongly decreased the accumulation of *Tos17* circular ecDNAs, measured by semi-quantitative PCR (Supplemental Figure 3B). Consistently, *Tos17* copy number (estimated by qPCR) was reduced by about 50% and 80% in calli generated in the presence of 20 and 40 µM Tenofovir, respectively, compared to calli generated in the absence of the drug (Figure 3A).

Circular ecDNA of LTR-TEs, a byproduct of LTR transposons activity, can be generated either by recombination of LTR-TE copies integrated in the genome or from linear ecDNA, and it is generally not reintegrated into the genome (Gaubatz, 1990). In contrast, linear LTR-TE ecDNA is a direct product of reverse transcription and can be inserted into the genome. Therefore, we used the de novo sequencing approach called ALE-seq (Cho et al., 2019) to detect LTR sequence of linear ecDNA. With this method, we detected a substantial number of reads corresponding to the *Tos17* linear LTR sequence in calli propagated on standard media, but virtually no reads in samples propagated in the presence of 40 μM Tenofovir (Figure 3B). This result was validated by semi-quantitative PCR with two sets of *Tos17* specific primers applied on the same cDNA used for sequencing (Supplemental Figure 3C, Materials and Methods). We also applied this approach to *Tos19*, a different LTR transposon which is also active in rice calli (Sabot et al., 2011), and we obtained a pattern very similar to what was observed for *Tos17* (Figure 3B). These results indicate that Tenofovir application to rice calli can efficiently reduce levels of linear ecDNA of the two most active LTR-TEs mobilized by in vitro tissue culture.

Finally, to directly evaluate the efficiency of Tenofovir to restrict LTR-TE transposition during the entire process of plant regeneration from tissue culture, we used an established protocol (Nishimura et al., 2006) to induce shoots and regenerate plants from Tenofovir-treated and control rice calli. We regenerated eight, five, and one plants from 36 initial calli induced, respectively, in the presence of 0, 20, and 40 µM Tenofovir (12 calli initialized per treatment) (Supplemental Figure 3A). Although the highest Tenofovir concentration used (40 µM) might have reduced the regeneration rate, all regenerated plants developed normally and did not display alterations until the reproductive stage, independently of the concentration of Tenofovir in the original media (Figure 3C).

Transposon display showed that none of the six plants we regenerated from calli exposed to Tenofovir had new *Tos17* insertions (Figure 3D). In contrast, we observed an average of 3.25 of new *Tos17* insertions per genome in the eight plants regenerated from calli propagated in standard medium, with all plants but one showing new *Tos17* integrations (Figure 3D). By cloning and sequencing of new junction fragments we verified that at least in one plant (plant #3, Figure 3D) *Tos17* had been inserted at new chromosomal locations, disrupting the coding sequences of two genes (Supplemental Figure 3, D and E). These results indicate that the application of 20 µM Tenofovir in the culture medium is likely to allow the regeneration of rice plants free from new LTR-TE insertions.

## Discussion

It is likely that LTR-TEs mobilization leads to phenotypic diversity that could be exploited to develop new crop varieties (Paszkowski, 2015). Nevertheless, uncontrolled TE mobilization may also have mutagenic deleterious effects. Hence, LTR-TEs can be a valuable tool in plant breeding but only if their activity is under strict control. So far, the application of DNA methyltransferase inhibitors has been efficiently used to induce epigenetic changes and activate LTR-TEs in plants (Griffin et al., 2016; Thieme et al., 2017; Catoni and Cortijo, 2018; Roquis et al., 2021). In contrast to these chemicals, Tenofovir is a drug with a specific inhibitory effect on LTR-TE mobilization, acting with a mechanism which is independent on the alteration of RNA silencing or associated epigenetic mechanisms. We found that the Tenofovir action is independent from the production of LTR-TE mRNA (Figure 1), suggesting that this drug could be used to discriminate specific effects of LTR-TE ecDNA accumulation directly. Therefore, Tenofovir has also the potential to be a valuable tool to study LTR-TE regulation in various biological models.

Contrary to Tenofovir, we did not observe *ONSEN* suppression for the other RT inhibitors tested, and this could reflect different molecular proprieties of Tenofovir (a nucleotide analogous) compared to nucleoside and non-nucleoside RT inhibitors. For example, it is known that nucleotide RT inhibitors cannot be cleaved by esterases and are therefore more resistant to DNA repair mechanisms (De Clercq, 2009). Nonetheless, it is also possible that Tenofovir is assimilated into roots and systemically mobilized in plant tissue more efficiently compared to the other chemicals, and we cannot exclude those other drugs could also effectively suppress LTR-TE mobilization if delivered with different methods or in different experimental conditions.

In Arabidopsis, the mutations of epigenetic factors such as the main DNA methyltransferase MET1 or the chromatin remodeler DDM1 produce genome-wide DNA demethylation and bursts of TE mobilization. This leads to phenotypic abnormalities and transgenerational decrease of fitness (Mirouze et al., 2009; Tsukahara et al., 2009; Griffiths et al., 2018). Interestingly, these effects remain mostly evident in crosses of MET1 and DDM1 mutants with isogenic wild-type lines, and in their segregating wild-type progenies (Catoni and Cortijo, 2018). Specifically, several TEs were found to remain mobile for up to eight generations following the re-establishment of wild-type alleles in epigenetic recombinant inbred lines obtained from one *met1* or *ddm1* original parent (Catoni et al., 2019; Quadrana et al., 2019). Therefore, Tenofovir could be also used to discriminate the role of LTR-TEs mobilization from other TEs or epialleles, in relation to genome instability observed in hypomethylated plant genomes.

Although several protocols for crop transformation and genome editing are available, they all require growing plant tissue in vitro as a necessary step in the regeneration of plants starting from genetically modified cells. Therefore, the uncontrolled LTR-TE mobilization induced during tissue culture makes it challenging to demonstrate “substantial equivalence” of new varieties obtained in this way with the natural antecedent cultivars, a process which is necessary to commercialize a new crop variety (Millstone et al., 1999; Gao et al., 2009; Li et al., 2019). In this context, the application of Tenofovir in the tissue culture media could become a very valuable approach to reduce the genetic diversity in propagated plant material, by inhibiting LTR-TE mobilization and facilitating the regeneration of plants genetically more uniform. Tenofovir suppression is expected to occur only for TEs that use RT enzymes for their transposition, while it will not affect somaclonal variation caused by other transposons (i.e. DNA transposons), sequence rearrangements, or changes in epigenetic state. Although rice is virtually the only major crop where LTR-TE activation during micropropagation has been extensively described, there are reports of LTR-TE mobilization occurring in tissue culture of other plant species, including tobacco (*Nicotiana tabacum*) (Hirochika, 1993), sweet potato (*Ipomoea batatas*) (Tahara et al., 2004), carrot (*Daucus carota*) (Kwolek et al., 2022), *Medicago truncatula* (d’Erfurth et al., 2003), bamboo (*Phyllostachys edulis*) (Zhou et al., 2018), and date palm (*Phoenix dactylifera*) (Mirani et al., 2020). Considering that for many crops the genetic resources are still limited, it could be possible that LTR-TE mobilization during plant regeneration has been mostly overlooked. However, considering the increasing availability of plant genome reference assemblies and TE detection tools (Satheesh et al., 2021), it is likely that LTR-TE activation will be documented in even more species, and Tenofovir will have application going beyond the stable in vitro propagation of Arabidopsis and rice.

The RT inhibitors have well-documented antiretroviral activity (Deeks et al., 1998; Maenza and Flexner, 1998), and Tenofovir is commonly used in therapies against HIV and hepatitis B virus infections (De Clercq, 2009; Wassner et al., 2020). Yet, the possible effect of RT inhibitors on TE mobilization is poorly investigated even in animal models (Jones et al., 2008; Banuelos-Sanchez et al., 2019), and in plants, Tenofovir was previously tested only as a potential antiviral agent against pathogenic virus infections (Špak et al., 2011). This work provides evidence that Tenofovir could be used efficiently to generically suppress the activity of RT enzymes encoded by LTR-TEs. Consequently, it is possible that the use of RT suppressors, such as Tenofovir or related molecules, will become a standard approach to suppress LTR-TE mobilization in multiple models, which might include plants, fungi, protozoan, and animals, facilitating studies related to genome plasticity and evolution in multicellular organisms.

## Materials and methods

### Screening for chemical inhibition of retrotransposition using Arabidopsis *ONSEN* activation by heat

All Arabidopsis (*A. thaliana*) plants used were in Col-0 background. The *nrpd1a-3* mutant line used was described previously (Herr et al., 2005). Seedlings were grown on ½ Murashige and Skoog (MS) media (1% w/v sucrose and 0.8% w/v agar, pH 5.7) at 20°C under 12 h light conditions (12 h light/12 h dark). In drug-treated plants, Tenofovir (Cayman Chemical, N 13874), Nevirapine (Merck, SML0097), Stavudine (Merck, Y0000408), Lamivudine (Merck, L1295), or Zidovudine (Stratech Scientific Ltd, B2221) were dissolved in dimethyl sulfoxide (DMSO) and added to the medium at the final concentration specified. The same volume of DMSO was added to the control untreated medium. Seven days after germination, plants were treated to activate *ONSEN*, as described previously (Ito et al., 2011) (Supplemental Figure 2A). One-week-old seedlings were primed by cold treatment at 24°C and immediately transferred to normal growth conditions (control plants or CS) or to 37°C for 24 h (heat-stressed or HS) plants. Plants were collected 24 h after heat application (HS0) and accumulation of linear extrachromosomal DNA (ecDNA) of *ONSEN* was evaluated by qPCR as described before (Ito et al., 2011).

Tenofovir-treated and control Arabidopsis plants were subsequently grown at 21°C for the remaining time, and samples were collected 0, 3, 11, and 14 days after heat stress depending on the experiment (condition defined CS0, CS3, CS11, CS14, and HS0, HS3, HS11, HS14 for control and heat-stressed plants, respectively).

Depending by the experiment, pools of 15–20 seedlings (2 weeks post-germination) or individual plant leaves (150 mg), were harvested and flash-frozen in liquid nitrogen. Individual Arabidopsis seedlings were transferred to soil (Levington F2) and grown until the flowering time. Progeny of these plants was used to test for the presence of new insertions by Transposon Display.

### Genome-wide expression analysis

Arabidopsis plants were grown and treated as described above. Pools of 15–20 plants were harvested and flash-frozen in liquid nitrogen at specific time points (CS0, CS0 + T, HS0, HS0 + T, HS3, HS3 + T). Three pool replicates were collected from each condition.

Total RNA was isolated from seedlings using the RNeasy Plant Mini Kit (Qiagen), following the manufacturer's instructions. The RNA quality and integrity were assessed on the Agilent 2200 Tape Station. Library preparation was performed using 1 mg of high-integrity total RNA (RIN > 8) using the TrueSeq Stranded mRNA Sample Prep Kit (Illumina, San Diego, CA). Libraries were sequenced in-house on an Illumina NextGen 500 sequencer using paired-end sequencing of 150 bp in length.

### RNA-seq mapping and differential expression analysis

The raw reads obtained from sequencing were analyzed using a combination of publicly available software and in-house scripts. Trimmomatic (Bolger et al., 2014) was used to remove adapters and discard low-quality reads. The cleaned reads were mapped to the Arabidopsis reference genome (TAIR10 version) by TopHat2 (Kim et al., 2013). Picard tools (available from http://broadinstitute.github.io/picard/) were used to discard duplicated reads. The processed data were visualized in SeqMonk (version 0.32.1 available from www.bioinformatics.babraham.ac.uk). The reads were quantitated and clustered using the RNA-seq Quantitation pipeline integrated into SeqMonk. Mapped reads from TopHat2 analysis were counted using htseq-count (Anders et al., 2015), and the raw counts per gene or per TE (TAIR 10 annotation) were used to determine differentially expressed features between the control and Tenofovir-treated samples with DESeq (Anders and Huber, 2010), using the default parameters. Genes or TEs with a significance *P*-value below 0.05 after Benjamini and Hochberg correction were considered to be differentially expressed. DEGs were used for analysis of GO enrichment using agriGO, a GO analysis toolkit (Du et al., 2010). Hierarchical clustering of the full transcriptomics of the samples was undertaken using the SeqMonk tool. The correlation between transcriptomic profiles of genes and TEs was calculated using the averaged values of fragments per kilobase of exon per million mapped fragments (FPKMs) of three biological replicates, for samples untreated and treated with Tenofovir, and was visualized as scatter plots in an R programming environment. Pearson correlation coefficient was calculated using R/Bioconductor (v3.8).

### Procedure of induction of Rice calli and regeneration of the plants

Rice (*O. sativa*) calli were induced by the method used for rice transformation as previously described (Nishimura et al., 2006). Briefly, seeds of *O. sativa ssp. japonica cv. Nipponbare* were surface-sterilized in 20% bleach for 15 min, rinsed three times with sterile water, and placed on N6D media in the presence of Tenofovir. Compared to the Arabidopsis experiments, we increased Tenofovir concentration in the media, and we supplemented with Tenofovir at 0, 20, or 40 μM in the plates. This was done because the rice calli were to remain in tissue culture for a longer time, and also because we assumed that the assimilation of the drug in the calli was less efficient than in Arabidopsis seedlings. Plates were incubated at 27°C in the dark. The calli formed after 3–4 weeks and were kept on plates for at least another 4 weeks before plant induction. For shoot regeneration, calli were transferred to MS-NK medium and were kept in plates for around 8 weeks. Shoots of 3–4 cm in length were transferred in MS medium in plant boxes. Transplant rooted plantlets were then moved into soil pots, with one plantlet per pot (Nishimura et al., 2006). We managed to regenerate eight, five, and one plant from 36 initial calli induced in the presence of 0, 20, and 40 µM of Tenofovir, respectively (12 calli initialized per treatment) (Supplemental Figure 3A).

### Detection of ecDNA of *Onsen* and Tos17

Total DNA was extracted from Col-0 and *nrpd1-3* Arabidopsis plants or from rice calli grown with or without Tenofovir, using the plant DNeasy kit (Qiagen) according to the manufacturer's instructions. The presence of *ONSEN* and *Tos17* circular ecDNA was tested using inverse-PCR with primers designed inside LTRs in opposite directions (Supplemental Table 7). The PCR reaction was performed using GoTaq polymerase (Promega). The PCR conditions were as follows: (i) *ONSEN*—95°C 2 min, 35 cycles 95°C 30 s, 58°C 30 s, 72°C 2 min; (ii) *Tos17*—95°C 2 min, 35 cycles 95°C 30 s, 55°C 30 s, 72°C 30 s. PCR amplicons from actin and eEF-1a were used as loading controls for *ONSEN* and *Tos17*, respectively.

qPCR analysis of LTR-TE DNA was performed on a LightCycler480 (Roche) using LightCycler 480 SYBR Green Master (Roche) with three technical replicates for each sample. For *ONSEN*, the relative copy number was calculated using the actin 2 gene and C-REPEAT/DRE BINDING FACTOR 2 as reference and normalized by DNA levels in Col-0 collected at CS0. For *Tos17*, the relative copy number of circular extrachromosomal DNA was calculated using Ubiquitin as a reference and normalized by DNA levels measured in the samples collected from untreated calli. Primers used are listed in Supplemental Table 7.

### Transposon display

Transposon display was applied as described previously with minor modifications (Griffiths et al., 2018). GenomeWalker (GW) adaptor oligos were prepared at 25 µM (Supplemental Table 7) and then heated at 95°C for 10 min and allowed to cool to room temperature. Genomic DNA was extracted from seedlings using the Cetrimonium bromide method; 300 ng DNA was digested using the *Dra1* restriction enzyme (New England Biolabs), which produced blunt-ended fragments compatible for ligation with the GW adaptor (300 ng DNA, 5 µL cut smart buffer, 2.5 µL Dra1, and H20 to 50 µL). Digestion was carried out at 37°C for 16 h, cleaned using a QIAGEN Gel Extraction Kit, and eluted in 2 × 15 µL EB buffer. Adaptor ligation was carried out at 16°C for 24 h (5 µL digested DNA, 2 µL GW adaptors (25 µM), 1.6 µL 10x ligation buffer, 1 µL T4 Ligase, and H_2_O to 16 µL). Ligated DNA was diluted 1:20. To detect new insertions of *ONSEN*, PCR was carried out using a primer matching the adaptor sequence (GenomeWalker_AP1) and a primer matching the LTR sequence (TD_ONSEN_LTR). In the case of *Tos17*, nested PCR was carried out using two primers matching the adaptor sequence (GenomeWalker_AP1 and GenomeWalker_AP2) paired with two primers matching *Tos17* LTR (TD_TOS17_LTR_1, and TD_TOS17_LTR_2). All primer sequences are reported in Supplemental Table 7. PCR was carried out using GoTaq polymerase (Promega) in the following conditions: primary PCR 95°C 2 min, 33 cycles 95°C 30 s, 58°C 30 s, 72°C 1 min; secondary PCR on 1:100 dilution of primary PCR 95°C 2 min, 35 cycles 95°C 30 s, 58°C 30 s, 72°C 1 min. PCR products were cut from the gel and ligated into pGEM-T for sequencing. The fragment sequences are reported in Supplemental Figure 3D.

### ALE-seq and targeted LTR detection

Genomic DNA was extracted from 100 mg of callus samples using a DNeasy Plant Mini Kit (Qiagen) following the manufacturer's instruction, and 100 ng was used for ALE-seq following the previously described protocol (Cho et al., 2019) with minor modifications. An adapter containing T7 promoter sequence at its 5′ end, was ligated to the end of extrachromosomal DNA, followed by in vitro transcription with T7 RNA polymerase. The synthesized RNA was then reverse transcribed using the primer binding the transcripts at the LTR-TE conserved primer-binding site (PBS). At this stage, to specifically amplify *Tos17* LTR, the cDNA was PCR-amplified using PBS and *Tos17* specific primers (Supplemental Table 7), and the PCR product was loaded onto an agarose gel. PCR was carried out using GoTaq polymerase (Promega) and with the following conditions: 95°C 2 min, 30 cycles 95°C 30 s, 55°C 30 s, 72°C 30 s. The remaining cDNA was amplified following the ALE-seq procedure (Cho et al., 2019), and strand specific sequencing was performed using Illumina MiSeq platform and 300 bp reads.

### Insertion confirmations in rice

To confirm the presence of new *Tos17* insertions in regenerated rice plants (plant #3, Figure 3D), the genomic DNA extracted as described above was used for PCR performed using primer pairs including a transposon-specific primer (*Tos17*) and a primer located in the flanking region of the new insertion (the loci LOC_Os07g35610 and LOC_Os11g10510). Primer sequences are reported in Supplemental Table 7. The PCR products were then run and extracted from the gel, and then ligated into pGEMT-easy (PROMEGA) to confirm their DNA sequence by Sanger sequencing.

### Accession numbers

Sequencing data have been deposited in Gene Expression Omnibus under the accession numbers GSE196868 and GSE196869. Gene accession numbers: LOC_Os07g35610, LOC_Os11g10510, AT1TE12295 (*ONSEN*).

## Supplemental data

The following materials are available in the online version of this article.

**Supplemental Figure S1**. Screening for chemicals inhibiting activation of retrotransposition in plants.

**Supplemental Figure S2**. Tenofovir treatment does not significantly affect plant growth and response to heat stress.

**Supplemental Figure S3**. Tenofovir inhibits *Tos17* mobilization in rice calli.

**Supplemental Table S1**. Summary of RNA-seq metrics.

**Supplemental Table S2**. Summary of RNA-seq results (FPKM).

**Supplemental Table S3**. Summary of TE expression analysis (FPKM).

**Supplemental Table S4**. List of DE genes in Tenofovir-treated Arabidopsis samples.

**Supplemental Table S5**. GO enrichment in DE genes due to Tenofovir treatment in control conditions (CS0+/−T).

**Supplemental Table S6**. GO enrichment in DE genes due to Tenofovir treatment in samples recovering from stress (HS3+/−T).

**Supplemental Table S7**. List of primers/oligoes used for RNA-seq validations.

## Supplementary Material

kiac605_Supplementary_Data
