# Supplementary material for: Specific suppression of long terminal repeat retrotransposon mobilization in plants
Source: Plant Physiol. 2022 Dec 30;191(4):2245–55. doi: 10.1093/plphys/kiac605 (PMC10069891; doi:10.1093/plphys/kiac605)
Supplement: kiac605_Supplementary_Data [file kiac605_supplementary_data.zip › Tenofovir_paper_supplementary material_v4.pdf]

Supplementary Material

Specific suppression of LTR retrotransposon mobilisation in plants.

Anna Brestovitsky[1], Mayumi Iwasaki[1,2], Jungnam Cho[1,3], Natthawut Adulyanukosol[1],

Jerzy Paszkowski[1], Marco Catoni[1,4,5]

[1] The Sainsbury Laboratory, University of Cambridge, Cambridge, UK.
[2] Department of Plant Biology, University of Geneva, Geneva, Switzerland.
[3] CAS Center for Excellence in Molecular Plant Sciences, Chinese Academy of Sciences, Shanghai, China.
[4] School of Biosciences, University of Birmingham, Birmingham, UK.
[5] Institute for Sustainable Plant Protection, National Research Council of Italy, Torino, Italy

Supplemental Tables:

Supplemental Table S1. Summary of RNA-seq metrics
Supplemental Table S2. Summary of RNA-seq results (FPKM)
Supplemental Table S3. Summary of TE expression analysis (FPKM)
Supplemental Table S4. List of DE genes in Tenofovir-treated Arabidopsis samples
Supplemental Table S5. GO enrichment in DE genes due to Tenofovir treatment in control conditions (CS0+/-T)
Supplemental Table S6. GO enrichment in DE genes due to Tenofovir treatment in samples recovering from stress (HS3+/-T)
Supplemental Table S7. List of primers/oligoes used for RNA-seq validations

Supplemental Figures:

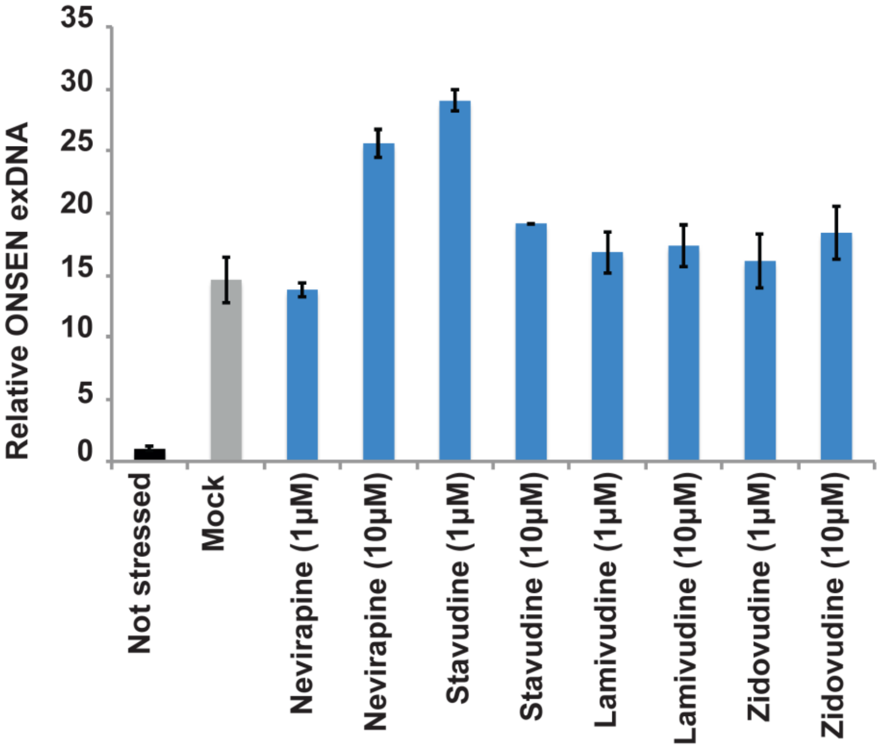

Supplemental Figure S1. Screening for chemicals inhibiting activation of retrotransposition in plants.

Arabidopsis *nrpd1* seedlings grown in the presence of chemical inhibitors of Reverse Transcriptase (Nevirapine, Stavudine, Lamivudine, and Zidovudine) at two concentrations in the medium (1 and 10 µM). The plants were heat stressed for 24 hours at 7 days after germination, and accumulation of ecDNA of *ONSEN* was evaluated by qPCR. Unstressed mutant seedlings (marked as not stressed) and heat stressed seedlings without drugs (Mock) were used as control. Each bar represents the mean of three technical replicates; ±s.d. marked by error bars.

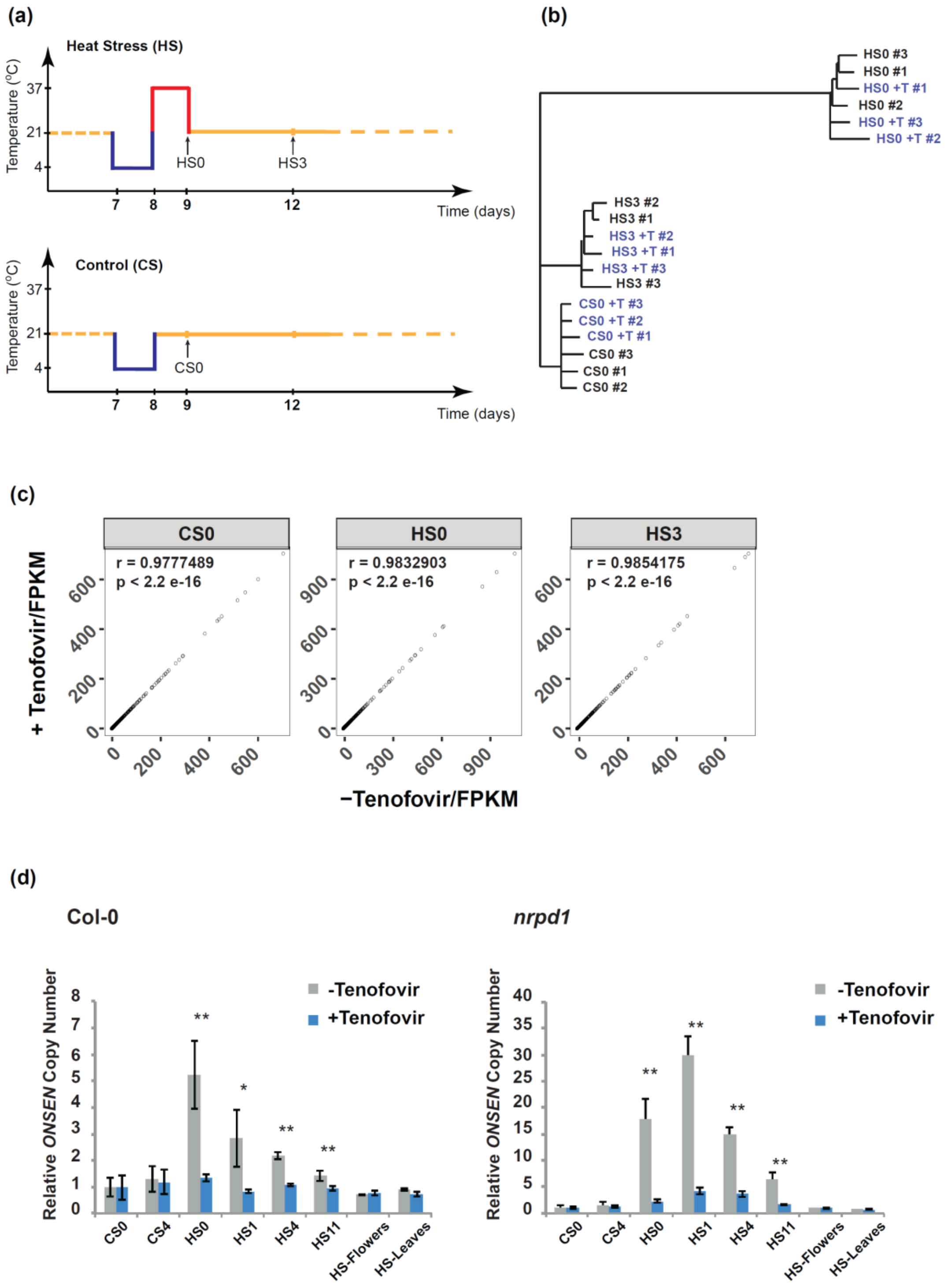

Supplemental Figure S2. Tenofovir treatment does not significantly affect plant growth and response to heat stress.

a) Schematic representation of the heat stress experiment. One-week old Arabidopsis seedlings were incubated at 4°C for 24 hours and immediately transferred to optimal growth conditions (Control plants, marked as CS) or to 37°C for 24hr (Heat Stressed, marked as HS) to induce *ONSEN* activation (Ito et al., 2011). Then, control and heat stressed plants were grown at constant 21°C, and samples were collected

immediately (0 days) and 3 days after heat-stress (generating the samples CS0, HS0 and HS3).
b) Hierarchical Clustering of the full transcriptomic profiles of samples treated as described in Fig. S2a. In blue are indicated the samples treated with Tenofovir.
c) Scatter plots summarizing correlation of TE expression in plant samples grown in absence or in presence of Tenofovir (10 µM). The data are from not stressed plants (CS0), plants collected after heat stress (HS0) and three days after the stress was applied (HS3). Each dot represents a single gene. Pearson correlation was used to assess the significance of the gene expression differences (r and p-value are indicated).
d) Relative copy number of *ONSEN* in Col-0 (left panel) and *nrpd1-3* (right panel) seedlings grown in absence or in presence of Tenofovir (10 µM), calculated using the Actin 2 (*ACT2*) and C-REPEAT/DRE BINDING FACTOR 2 (*CBF2*) genes as reference. Values displayed are relative to the *ONSEN* DNA levels in Col-0 collected at CS0. Each bar represents the mean of three repetitions; ±s.d. marked by error bars. P-values were calculated by a one-tailed Student's t-test. * = p < 1x10-2; ** = p < 1x10-3.

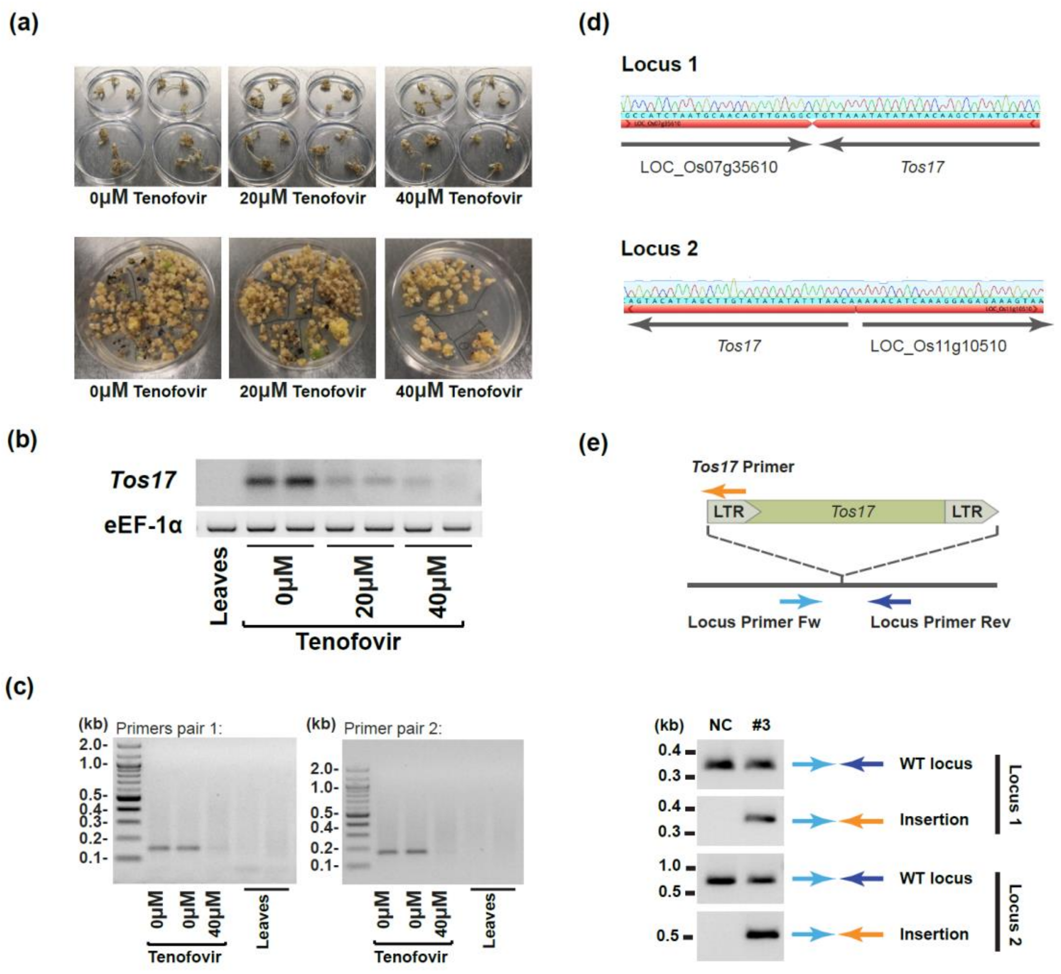

Supplemental Figure S3. Tenofovir inhibits *Tos17* mobilisation in rice calli.

a) Representative pictures of generated rice calli at the stages of 3 weeks (upper panels) and 7 weeks (lower panels) following the induction. The calli were grown in media containing 0, 20 or 40 µM of Tenofovir, as indicated.

b) Detection of *Tos17* circular ecDNA forms accumulated during rice callus induction in absence (0 µM) or in presence (20 µM and 40 µM) of Tenofovir. PCR of eEF1α gene is used as loading control.

c) Accumulation of linear ecDNA of *Tos17* extrachromosomal DNA from rice calli generated in absence (0 µM) and in presence (40 µM) of Tenofovir. The *Tos17* ecDNA was amplified from cDNA obtained by ALE-seq using two different pairs of *Tos17* specific primers (left and right panels) (Primer sequences are reported in **Table S7**). DNA from wild type leaves was used as negative control.

d) Scheme representing the sequence of two new *Tos17* insertions in coding genes, obtained by cloning and sequencing the Transposon Display product from lane#3 in the **Fig. 3d**. The figure displays the region joining *Tos17* LTR and the genes neighbouring the new integration. The scheme was obtained with the program Geneious (Biomatter).

e) Validation of two new *Tos17* insertions in plants regenerated from calli grown in absence of Tenofovir (sample #3 in the **Fig. 3d**). Total DNA from wild type leaves was used as negative control (marked as NC). Location of the primers used for PCR validation is shown.
